# AgNPs Argovit™ Modulates Cyclophosphamide-Induced Genotoxicity on Peripheral Blood Erythrocytes In Vivo

**DOI:** 10.3390/nano11082096

**Published:** 2021-08-18

**Authors:** Idalia Yazmin Castañeda-Yslas, Olivia Torres-Bugarín, Juan Carlos García-Ramos, Yanis Toledano-Magaña, Patricia Radilla-Chávez, Nina Bogdanchikova, Alexey Pestryakov, Balam Ruiz-Ruiz, María Evarista Arellano-García

**Affiliations:** 1 Programa de Maestría y Doctorado en Ciencias e Ingeniería (MyDCI), Facultad de Ciencias, Universidad Autónoma de Baja California, Ensenada 22860, Baja California, Mexico; idalia.castaneda@uabc.edu.mx; 2 Departamento de Ciclo de Vida y Medicina Interna II, Decanato Ciencias de la Salud, Universidad Autónoma de Guadalajara, Zapopan 45129, Jalisco, Mexico; oliviatorres@hotmail.com; 3 Escuela de Ciencias de la Salud Unidad Valle Dorado, Universidad Autónoma de Baja California, Ensenada 22890, Baja California, Mexico; yanis.toledano@uabc.edu.mx (Y.T.-M.); patyradilla@uabc.edu.mx (P.R.-C.); 4 Centro de Nanociencias y Nanotecnología, Universidad Nacional Autónoma de México, Ensenada 22860, Baja California, Mexico; nina@cnyn.unam.mx; 5 Research School of Chemistry and Applied Biomedical Sciences, Tomsk Polytechnic University, 634050 Tomsk, Russia; pestryakov2005@yandex.ru; 6 Departamento de Ciencias de la Salud, Unidad Regional Los Mochis, Universidad Autónoma de Occidente, Los Mochis 81223, Sinaloa, Mexico; balam.ruiz@uadeo.mx; 7 Facultad de Ciencias, Universidad Autónoma de Baja California, Ensenada 22860, Baja California, Mexico

**Keywords:** silver nanoparticles, genotoxicity, genotoxic modulation, antineoplastic agents, cyclophosphamide, peripheral blood erythrocytes, micronuclei

## Abstract

Silver nanoparticles (AgNPs) have been studied worldwide for their potential biomedical applications. Specifically, they are proposed as a novel alternative for cancer treatment. However, the determination of their cytotoxic and genotoxic effects continues to limit their application. The commercially available silver nanoparticle Argovit™ has shown antineoplastic, antiviral, antibacterial, and tissue regenerative properties, activities triggered by its capacity to promote the overproduction of reactive oxygen species (ROS). Therefore, in this work, we evaluated the genotoxic and cytotoxic potential of the Argovit™ formulation (average size: 35 nm) on BALB/c mice using the micronucleus in a peripheral blood erythrocytes model. Besides, we evaluated the capability of AgNPs to modulate the genotoxic effect induced by cyclophosphamide (CP) after the administration of the oncologic agent. To achieve this, 5–6-week-old male mice with a mean weight of 20.11 ± 2.38 g were treated with water as negative control (Group 1), an single intraperitoneal dose of CP (50 mg/kg of body weight, Group 2), a daily oral dose of AgNPs (6 mg/kg of weight, Group 3) for three consecutive days, or a combination of these treatment schemes: one day of CP doses (50 mg/kg of body weight) followed by three doses of AgNPs (one dose per day, Group 4) and three alternate doses of CP and AgNPs (six days of exposure, Group 5). Blood samples were taken just before the first administration (0 h) and every 24 h for seven days. Our results show that Argovit™ AgNPs induced no significant cytotoxic or acute genotoxic damage. The observed cumulative genotoxic damage in this model could be caused by the accumulation of AgNPs due to administered consecutive doses. Furthermore, the administration of AgNPs after 24 h of CP seems to have a protective effect on bone marrow and reduces by up to 50% the acute genotoxic damage induced by CP. However, this protection is not enough to counteract several doses of CP. To our knowledge, this is the first time that the exceptional chemoprotective capacity produced by a non-cytotoxic silver nanoparticle formulation against CP genotoxic damage has been reported. These findings raise the possibility of using AgNPs as an adjuvant agent with current treatments, reducing adverse effects.

## 1. Introduction

The increasing use of silver nanoparticles (AgNPs) in many fields and especially in biomedical applications requires the expansion of studies on their biocompatibility and biosecurity, especially concerning cytotoxicity and genotoxicity [1]. Research about the toxicity of different AgNPs continues to increase and uses a great diversity of biological models such as mice, plants, and cell cultures, generating controversy surrounding which are the most suitable models to detect cytotoxicity [2,3,4,5]. Some molecular processes induced by different AgNPs types have been clarified, such as DNA damage caused by reactive oxygen species (ROS) [6]. However, controversy persists regarding the understanding of the action mechanisms of nanoparticles in biological systems [7,8] and how they interact with other drugs such as antibiotics, antiviral, and antineoplastic agents that could give them new therapeutic properties [9,10]. 

Since 2004, Argovit™, a commercial formulation of silver nanoparticles, has been studied in vitro and in vivo for its multiple biomedical applications (antiviral, bactericide, re-epithelialization agent, among others) [11,12,13,14]. The murine models used thus far do not show toxic effects on immune cells or organs [15]. This specific AgNP formulation showed cytotoxic effects in eight human cancer cell lines, apparently through the generation of ROS [16]. Previously reported results showed low toxicity in classic models like cytokinesis block micronucleus assay (CBMN) and the *Allium* test [17,18]. In the first case, no evidence of genotoxic or cytotoxic damage with Argovit™ concentrations of 0.012 to 12 μg/mL was observed on human lymphocytes. Besides, in the *Allium cepa* model, a concentration range of 5–100 μg/mL did not induce cytotoxic or genotoxic damage. It was suggested that the lack of damage was due to the polyvinylpyrrolidone (PVP)/silver ratio in the formulation. The promising results obtained with Argovit™ demonstrate the need for the completion of its toxicity studies.

In this context, an in vivo micronucleus test is commonly used to assess and identify genetic material damage produced by several materials and substances that do not necessarily kill the cells [19]. Micronuclei assay in peripheral blood erythrocytes (MNPBE) is one of the best methods to evaluate the number of mature and immature erythrocytes and the presence of micronucleus in these cells [20]. This technique provides a significant advantage directly applicable to environmental impact effects, work exposure, or health/disease monitoring by the generation of genetic material damage data over time, compared to other in vitro genotoxic methods which provide information of a specific time [19].

The antineoplastic compound cyclophosphamide is widely used as a positive control of myelosuppression (cytotoxicity) and irreversible DNA damage (genotoxicity) in the micronucleus assay in mouse peripheral blood erythrocytes [21,22]. Its toxicity is dose-dependent, generates micronucleus and myelosuppression, reduces the number of erythrocytes [23], and is not specific to cancer cells [24,25,26].

Therefore, the present work explores the cytotoxicity and genotoxicity of Argovit™ AgNPs based on the micronucleus technique in peripheral blood erythrocytes of BALB/c mice. The Argovit™ AgNP formulation was administered under different treatment schemes that include combination with the antineoplastic agent cyclophosphamide to identify its toxicity and protective potency.

## 2. Materials and Methods

### 2.1. AgNPs and Chemical Compounds

#### 2.1.1. Physicochemical Properties of AgNPs

Argovit™ AgNPs were donated by the Scientific and Production Center Vector Vita (Novosibirsk, Russia), and their physicochemical characteristics have been previously reported by our group [17]. The metallic silver particles are spheroids, and their sizes are within the range of 1 to 90 nm with an average size of 35 nm. Argovit™ AgNPs are coated with 18.8% (*w/w*) of PVP, the concentration of metallic silver is 1.2% (*w/w*), and the rest of the formulation is distilled water. The surface plasmon resonance absorbance was found at 420 nm, with a zeta potential of −15 mV, and a hydrodynamic diameter of 70 nm [16]. The plasmon resonance at 421 ± 1 nm for the batch used in this work confirms the silver nanoparticle presence. We acquired the spectrum of the AgNPs suspension before mice administration. 

#### 2.1.2. Evaluated Compounds

- Cyclophosphamide (CP) was applied intraperitoneally (i.p.) as a positive control. The i.p. dose of 50 mg/kg guarantees the induction of MN [22,27].
- Distilled water (200 µL, i.p.).
- AgNPs (6 mg/kg) were orally administered considering the results observed in other antiviral and antitumor in vivo models [13,14,28].

The application of positive and negative controls through different administration routes is accepted for this type of study [29]. All doses were adjusted to mice weight.

### 2.2. Animals and Collected Samples

#### 2.2.1. BALB/c Mice

The study was carried out on male BALB/c mice of 5–6 weeks of age and a mean weight of 20.11 ± 2.38 g obtained from the Centro de Investigación Biomédica de Occidente (CIBO) of Instituto Mexicano del Seguro Social (IMSS), Guadalajara, Jalisco, Mexico. The animals were placed inside polycarbonate cages bedded with sawdust powder, were acclimated to the laboratory conditions for two weeks before the treatment application, and were fed ad libitum with standard rodent pellets and water. The mice management was carried out as established in the Technical Specifications for Production, Care, and Use of Laboratory Animals (SAGARPA, 1999). For all groups, clinical signs, including skin and fur appearance, eyes, membranes, conduct, respiratory pattern, the presence of nausea, vomiting, diarrhea, tremors, convulsions, and lethargy, were carefully registered throughout the experiment. Animals were weighed at the beginning and the end of the experiment. Obtained values were compared with averaged weight values reported by the provider according to the age of the animal.

#### 2.2.2. Work Groups

**Figure 1:**
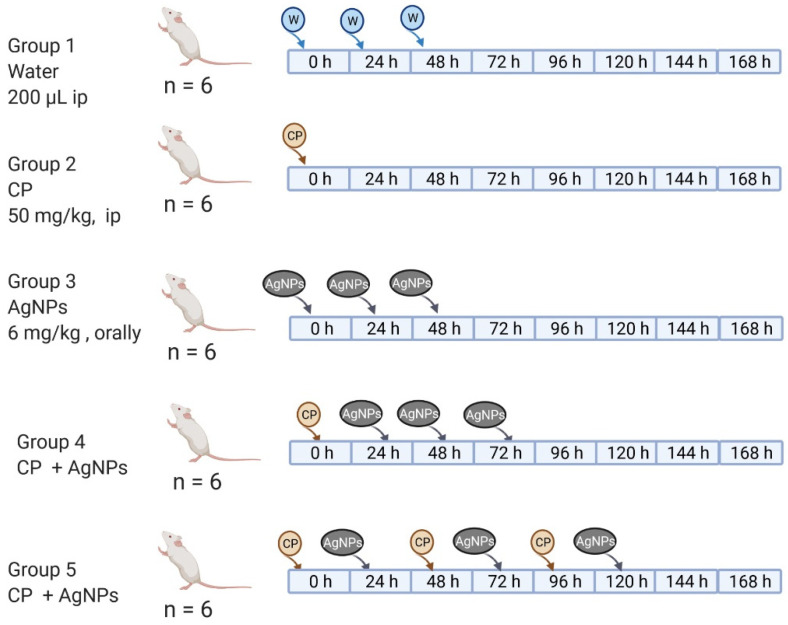
Administration schemes: Group 1: water (0, 24, 48), Group 2: CP (0 h), Group 3: AgNPs (0, 24, 48), Group 4: CP +AgNPs; CP (0 h) + AgNPs (24, 48, 72 h), and Group 5: CP +AgNPs; CP (0, 48, 96 h) + AgNPs (24, 72, 120 h).

Five random groups of six male mice were formed, as suggested by the OECD Guidelines for the Testing of Chemicals [25]. Before the first treatment, mice were weighed and marked non-invasively for later identification. Each group received specific treatment as shown in Figure 1. In all treatments, a single daily dose of AgNPs (6 mg/kg) or CP (50 mg/kg) was administered in the morning adjusted to mice body weight, as recommended by the OCDE guidelines [22]. At 168 h, all animals were sacrificed. 

#### 2.2.3. Sample Collection and Processing

**Figure 2:**
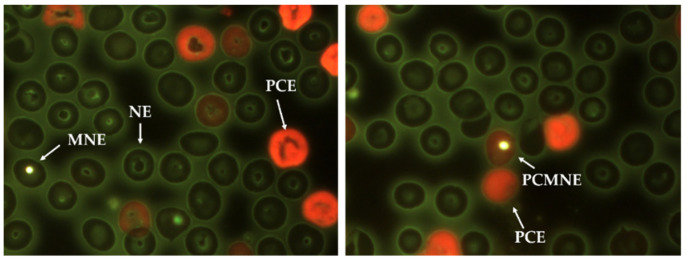
Representative images obtained for each biomarker. White arrows indicate normal erythrocytes (NEs), micronucleated erythrocytes (MNEs), polychromatic erythrocytes (PCEs), and micronucleated polychromatic erythrocytes (MNPCEs). Acridine orange staining, microscope Carl Zeiss^®^, 100× objective (Zeiss, Darmstadt, Germany).

Just before the first administration and during the treatment, a drop of peripheral blood from each mice tail were obtained each 24 h until 168 h, smeared on a sample holder, and air-dried for 10 min. After that, the sample was fixated with ethanol (80%, JT Backer, Phillipsburg, NJ, USA) for 10 min and then stained with acridine orange (Sigma-Aldich, St. Louis, MO, USA). All slides were air-dried and stored in the dark [30]. The preparations were examined with a fluorescence microscope (Axio Lab A1 Zeiss, Zeiss, Darmstadt, Germany), in 100× magnification with immersion oil. Figure 2 shows representative images used to perform the biomarker frequency of each group. Four biomarkers that enable the explanation of cytotoxicity and genotoxicity processes: normochromatic erythrocytes (NEs), polychromatic erythrocytes (PCEs, also called reticulocytes), micronucleated erythrocytes (MNEs), and micronucleated polychromatic erythrocytes (PCMNEs) were assessed. A total of 10,000 erythrocytes were analyzed per sample. Besides, PCE numbers were recorded in 1000 total erythrocytes (PCEs/1000 TE), the micronucleated polychromatic erythrocytes in 1000 polychromatic erythrocytes (MNPCEs/1000PCE), and the micronucleated erythrocytes in 10,000 total erythrocytes (MNEs/10,000 TE) [20].

### 2.3. Data Analysis

Statistical analysis was performed through two-way ANOVA and the post-hoc Tukey test (*p* < 0.0001) using Statistica 8.0 (StatSoft Inc., Hambuerg, Germany, 2008) software. The graphics were generated with GraphPad Prism 9.0.0 (GraphPad Software, San Diego, CA, USA, 5 November 2020).

## 3. Results

### 3.1. General Toxicity

The mice in this study assessed as per the administration schemes described in Section 2.2.2 showed no signs of toxicity throughout the assay. Compared with the control group, no weight loss, bristly hair, lack of mobility, or appetite changes were observed for the treated animals.

### 3.2. Myelosuppression-Cytotoxicity

PCEs are immature erythrocytes that have been present for less than a day in the bloodstream, with RNA residues can be seen orange with an acridine stain; if their frequency decreases significantly, this is due to myelosuppression by cytotoxic effect [31].

**Figure 3:**
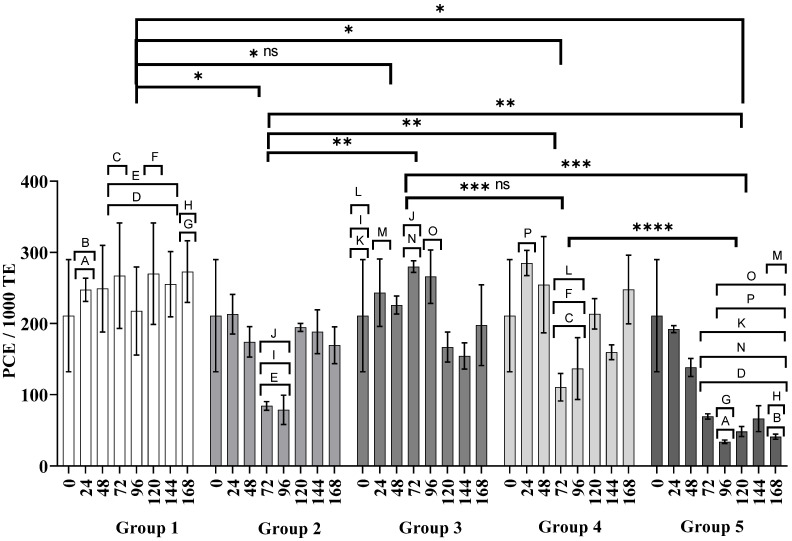
Polychromatic erythrocytes observed in 1000 total erythrocytes. Group 1: water (0, 24, 48 h), Group 2: CP (0 h), Group 3: AgNPs (0, 24, 48 h), Group 4: CP + AgNPs; CP (0 h) + AgNPs (24, 48, 72 h), and Group 5: CP + AgNPs; CP (0, 48, 96 h) + AgNPs (24, 72, 120 h). ns: no significant difference; *, **, ***, and **** denote statistically significant differences between groups (*p* < 0.0001); letters A–P denote statistically significant differences between inter and intragroup concentrations (*p* < 0.0001). Numeric data used to build the graph can be consulted in Table S1.

**Figure 4:**
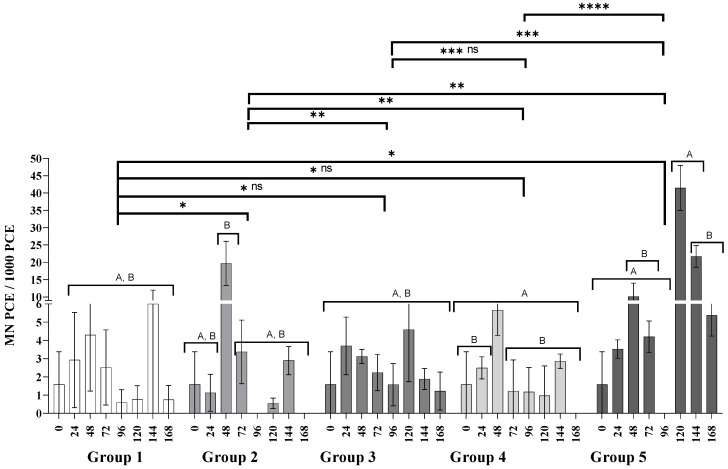
Micronucleated polychromatic erythrocytes observed in 1000 polychromatic erythrocytes. Group 1: water (0, 24, 48 h), Group 2: CP (0 h), Group 3: AgNPs (0, 24, 48 h), Group 4: CP + AgNPs; CP (0 h) + AgNPs (24, 48, 72 h), and Group 5: CP +AgNPs; CP (0, 48, 96 h) + AgNPs (24, 72, 120 h). ns: no significant difference; *, **, ***, and **** denote statistically significant differences between groups (*p* < 0.0001); letters A and B denote statistically significant differences between inter and intragroup concentrations (*p* < 0.0001). Numeric data used to build the graph can be consulted in Table S2.

In Figure 3 the frequency of PCEs over time can be seen, and it is noteworthy that in the water group (control) the PCE frequency did not change significantly over time (Group 1), in contrast, as expected in the CP group (Group 4), myelosuppression was observed because the frequency of PCEs decreased at 72 and 96 h (*p* < 0.0001, letter E on Figure 4), disappearing at 120 h. Regarding the group exposed to AgNPs (Group 3) the PCE frequency was similar over time to the water group. In the case of the administration of AgNPs 24 h after the administration of CP (Groups 4 and 5), these exhibited less myelosuppression than with a single dose of CP (Group 2), and at 120 h the bone marrow recovered. 

### 3.3. Genotoxicity

The micronuclei found in mature (MNE) and immature (MNPCE) erythrocytes are used as genotoxicity biomarkers [32]. MNE frequency is closely related to PCEs and must be interpreted in concomitance. The PCE frequency shown in Figure 4 exhibits the production of new erythrocytes or polychromatic erythrocytes recently incorporated into the bloodstream. The information provided in Figure 3 allows us to discern whether the frequency of micronucleated erythrocytes recorded in Figure 4 is derived directly from genotoxic damage or if there is interference by myelosuppression. 

In the case of AgNPs administered in three consecutive doses (Group 3), the frequency of MNPCE was similar over time, suggesting that the AgNPs are not genotoxic. Even when administered after the application of CP (Group 4), the nanoparticles show a cytoprotective effect since they reduce the acute genotoxic effect of CP, which is corroborated with the frequency of MNPCE being approximately 50% lower (*p* < 0.0001, letter E) than in organisms treated only with CP. However, the cytoprotective effect is not maintained when applying CP for more days, since the acute genotoxic damage is evident, as observed in Group 5, even with the myelosuppression observed in Figure 3.

Figure 4 shows the acute genotoxicity through the frequency of MNPCE that can only be scored within the first 24 h after its onset. It can be observed that, a single dose of CP produces a significant increase of MNPCE frequency after 48 h of the administration (19.7 ± 6.4); results similar to those reported by other authors [21,33]. On the other hand, the administration of three consecutive doses of AgNPs produce no acute genotoxic damage (Group 3). The administration of AgNPs seems to decrease the acute genotoxic effect of CP, since the MNPCE frequency registered at 48 h in the Group 4 mice is half of the registered frequency for Group 2 (5.7 ± 1.4 MNPCE/1000 TE; letter B, *p* < 0.0001, Figure 4). Acute genotoxic damage is evident in Group 5, where three alternate doses of CP followed by AgNPs 24 h later were administered. At 48 h the MNPCE count was 10.1 ± 3.9, with the most significant increase at 120 h with 41.5 ± 6.5 MNPCEs/1000 TE (Group 5, Figure 4, letter A *p* < 0.0001). The acute genotoxic damage in Group 5 is remarkable considering the myelosuppression observed since the 72 h point (Group 5, Figure 3).

**Figure 5:**
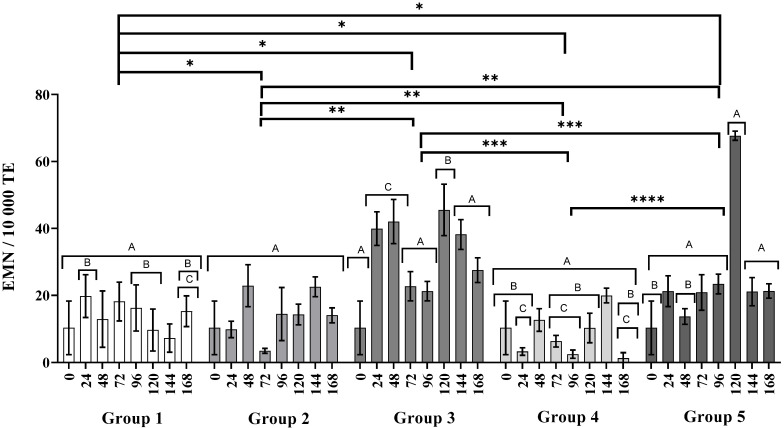
Micronucleated erythrocytes observed in 10,000 total erythrocytes. Group 1: water (0, 24, 48 h), Group 2: CP (0 h), Group 3: AgNPs (0, 24, 48 h), Group 4: CP+ AgNPs; CP (0 h) + AgNPs (24, 48, 72 h), and Group 5: CP +AgNPs; CP (0, 48, 96 h) + AgNPs (24, 72, 120 h). *, **, ***, and **** denote statistically significant differences between groups (*p* < 0.0001); letters A–C denote statistically significant differences (*p* > 0.0001) between inter and intragroup concentrations (*p* < 0.0001). Numeric data used to build the graph can be consulted in Table S3.

Figure 5 shows the cumulative genotoxic damage monitored by the frequency of MNEs in each group. No significant differences were observed in the frequency of MNEs between the groups treated with water (Group 1) and AgPNs (Group 3), and it should be noted that the frequency of MNEs was similar over time (*p* < 0.0001) in both.

The increase in MNE frequency observed at 24 and 48 h, despite noteworthy, was not taken into account on the genotoxic damage analysis following the recommendation of OECD guideline 474 [22]. On the other hand, the decrease in MNE frequency in the groups treated with three doses of AgPNs and one dose of CP observed at 96 and 120 h, respectively, is a phenomenon that could be due to the administration times of the drug, rather than the processes of myelosuppression and recovery of the bone marrow. 

## 4. Discussion

This work investigated the genotoxic potential of the commercially available Argovit™ AgNP formulation on BALB/c mice. This formulation has shown remarkable results as an antiviral [13,14,34,35], antimicrobial [36,37,38,39,40], and growth promoter of commercially relevant crops [41,42] with very low cytotoxic [17] and phytotoxic [18] damages. For the purposes of the study, we orally administered a daily dose of 6 mg/kg of AgNPs for three consecutive days and continued the observation for five more days. Blood samples from the tail vein were collected every 24 h to determine MNE, PCE, and MN-PCE frequencies throughout these eight days.

This study found no evidence of myelosuppression (Group 3, Figure 3) or acute genotoxic damage (Group 3, Figure 4) in BALB/c mice receiving three consecutive doses of 6 mg AgNPs/kg body weight. There is no doubt that the cellular uptake of PVP-AgNPs occurs on monocytes, lymphocytes, and erythrocytes. This process has been demonstrated by several techniques including transmission electron microscopy (TEM), phase-contrast microscopy, flow cytometric light-scattering, focused induced beam (FIB), scanning electron microscopy (SEM), and energy-dispersive X-ray spectroscopy (EDX) analysis [43,44,45]. Interestingly, accumulative genotoxic damage was observed 72 h after the last administration of AgNPs, although this damage decreased over time (Group 3, Figure 5). However, the hematopoietic system responded to the external stimuli increasing the production of erythrocytes (Group 3, Figure 4), which eventually decreased the MNE frequency over time (Group 3, Figure 4). This observation reflects the organism capacity to stabilize relatively quickly the activity of the bone marrow avoiding myelosuppression [46], suggesting no permanent damage of the bone marrow. 

As observed in Table 1, the genotoxic and cytotoxic effects of AgNPs are not clear, but the size of the nanoparticles, the metal content or type, and the quantity of coating agents could perform a fundamental role in terms of genotoxicity [2,47,48,49,50,51]. Table 1 shows that AgNPs bound to citrate or TiO_2_ induce DNA breaks and hence, the formation of MN [47]. On the contrary, formulations with PVP do not show cytotoxic or genotoxic damage; therefore, it is suggested that PVP coating seems a critical factor in the absence of genotoxicity of these particles. PVP coating contributes to the biodistribution of the AgNPs. Studies in rats have shown that 98% of this formulation is excreted in the feces [52,53], which helps to explain its low genotoxic effect. Unfortunately, the lack of complete physicochemical information regarding the silver content or PVP: Ag ratio does not allow us to conduct a more in-depth discussion on this topic.

The great stability of the Argovit™ formulation and its low cytotoxicity could be the reason why myelosuppression or acute genotoxic damage is not observed. Some 20 nm AgNPs formulations have been reported to compromise viability (>10 µg/mL) and enzymatic and non-enzymatic antioxidant systems (>0.4 µg/mL) of erythrocytes in a concentration-dependent manner, with the released silver ions being responsible for those effects [54,55]. Silver ion release from AgNP formulation is directly related with its stability [56]. Recently we described that the metal/coating agent ratio plays a significant role in the hemolytic potential of AgNP formulations. Specifically, for those formulations coated with PVP, it was found that the higher the amount of coating polymer, the lower its hemolytic potential. The hemolysis produced by 400 µg/mL of Argovit™ on human erythrocytes is negligible, meanwhile other PVP-AgNPs formulations produce more than 5% of hemolysis with half- or ten-times lower concentrations [57]. Similarly, the metal/PVP ratio gives the Argovit™ formulation greater biocompatibility against human peripheral blood lymphocytes, compared to a formulation of AgNPs of similar size but with a lower PVP content in its coating [17].

On the other hand, the administration of a single dose of CP produces remarkable myelosuppression (Group 2, Figure 3) and acute genotoxic damage (Group 2, Figure 4) after 48 h and 72 h, respectively. The accumulative genotoxicity could be masked by the low production of erythrocytes and the low efficiency of the reticuloendothelial system [20,58]. Several authors have already found this behavior for CP [33,59,60]. Interestingly, in this work we found that Argovit™ nanoparticles seems to reduce myelosuppression (Group 4, Figure 2) and up to 50% of the acute genotoxic damage caused by CP (Group 4, Figure 3). A similar response was observed when the alternate administration of CP and AgNPs was administered to mice from Group 5. Figure 2 shows that myelosuppression is not solved, although the acute genotoxicity at 48 h is almost half of that registered with a single CP dose (Group 5, Figure 4) without evidence of accumulative genotoxicity at that time (Group 5, Figure 5). However, the protective effect was not so effective for the continuous administration of CP, as MN, PCE, and MNE frequencies significantly increased at 120 h.

A new question arises: do AgNPs serve as adjuvants for CP? The antiproliferative activity of Argovit™ AgNPs against several human tumor cell lines has been previously reported [40,61]. Therefore, this suggests that the combination of AgNPs + CP could behave as a potent antiproliferative agent, significantly decreasing the genotoxic damage induced by CP potentially attributed to the action of AgNPs. The modulation of the CP genotoxic effect found here is similar to that found with carnosine administrated before CP, attenuating the genotoxicity and cytotoxicity of CP in mouse bone marrow [27]. These authors found a carnosine dose-dependent decreasing MNPCE frequency behavior. Additionally, the administration of *S. officinalis* extract through feeding for 7 days [62] results in an anticlastogenic effect decreasing the micronucleus frequency induced by an intraperitoneal single dose of CP (40 mg/kg body weight) 1.5 h after the last feeding of *S. officinalis.*

Several natural products have shown an ability to decrease MN frequency provoked by CP, although practically all compounds must be administered before CP to show their chemoprotective effect [63,64,65,66,67,68]. Others, such as water-soluble tannins and triterpenoid saponins, have shown effectiveness when administered at the same time as CP (co-treatment), but only a few examples show a CP protective effect post-treatment [69,70,71]. To our knowledge, this is the first time that the antigenotoxic activity of AgNPs has been reported within an in vivo model after 24 h of CP administration (i.p., 50 mg/kg).

The buffering capacity of Argovit™ against acute and accumulative genotoxic damage at earlier stages seems not sufficient to counteract consecutive doses of CP. However, it is important to remember that the chosen CP dose of 50 mg/kg used in this work guarantees an MNPCE increase. We found that a single dose of 6 mg/kg of the AgNP formulation can reduce by up to 50% the MNPCE frequency elicited by a single dose of CP, delaying MNPCE production after multiple dose administrations of CP of a genotoxic concentration. Although the administration of AgNPs cannot reverse the myelosuppression caused by CP, it helps to reduce nuclear damage in PCEs. More studies must be performed to identify the events that lead to accumulative genotoxic damage without evidence of myelosuppression and acute genotoxic damage. Additionally, further experiments are needed to recognize the administration scheme that could be effective as adjuvant on antitumor treatments.

Despite the accumulative genotoxic damage observed after three consecutive doses of AgNPs, the above results prove that bone marrow incurs no damage after the oral administration of an effective dose of AgNPs Argovit™, as no changes were presented on immature erythrocyte population compared with the negative control. Figure 3, Figure 4 and Figure 5 show that Argovit™ counteracts myelosuppression and the acute and accumulative genotoxic damage elicited by repeated doses of CP. These results provide evidence of the antigenotoxic effect of AgNPs against a highly cytotoxic and genotoxic agent such as CP, allowing the exploration of new applications. They also contribute to the development of safe nanomaterials with biomedical applications.

## 5. Conclusions

The Argovit™ silver nanoparticle formulation presents neither cytotoxic nor acute genotoxic damage in peripheral blood erythrocytes of BALB/c mice treated with a 6 mg/kg scheme every 24 h for three days. Although cumulative genotoxic damage was observed, the effect disappeared over time without decreasing the PCE frequency. These results indicate the significant biocompatibility of these AgNPs on the murine model BALB/c, compared with CP that induces myelosuppression. To our knowledge, this is the first time that the modulation of genotoxic damage produced by cyclophosphamide has been reported using a non-cytotoxic silver nanoparticle formulation. Argovit™ can reduce by up to 50% the micronucleated polychromatic erythrocyte frequency (acute genotoxicity) when administered 24 h after a genotoxic dose of CP (50 mg/kg) and delay the appearance of micronuclei until 24 h after multiple cyclophosphamide administrations. These results suggest a silver nanoparticle-modulating effect on the damage that cyclophosphamide can generate in healthy cells. In addition to this genotoxic modulating effect, it seems that AgNPs reduce the cytotoxic damage caused by the previously applied antineoplastic agents.

These results will undoubtedly contribute to the design of new chemotherapy treatments that will diminish the adverse effects caused by the current treatments taking advantage of the exceptional antigenotoxic capacity of the Argovit™ silver nanoparticle formulation.

## Figures and Tables

**Table 1:** Cytotoxicity and genotoxicity studies of AgNPs with the micronucleus assay in rodent erythrocytes.

Model	Shape	Size (nm)	Hydrodynamic Diameter (nm)	Silver Content	Coating	Z Potential (mV)	Biomarker	Administration Scheme	Observed Effect	References
Mouse (male) BALB/c Five–six-weeks-old	Spheroidal	35	75	1.2% *w/w*	PVP	−15	MN peripheral blood erythrocytes	OA 6 mg/kg/daily/3 days	Neither cytotoxic nor acute genotoxic damage. Cumulative genotoxicity	This work
Mouse (C57BL/6J p^un^/p^un^)	ND	32 ± 0.7	ND	ND	PVP	ND	Oxidative damage MN frequency staining with Giemsa	OA 4 mg/kg daily/7 days	Neither oxidative damage nor MN frequency increase	[2]
Rats Wistar 14-weeks-old	Spheroidal	20 ± 5	20–77.29 ± 1.4	ND	ND	33.6	MN bone marrow	IV single dose AgNPs 20 nm: 5 and 10 mg/kg AgNPs 20 nm: 10 mg/kg	Genotoxicity after 24 h of exposure (*p* < 0.5)	[47]
		200 ± 50	200–333.12 ± 2.5	ND	ND	37.5		IV single dose AgNPs 200 nm: 5 mg/kg	Genotoxicity after 24 h of exposure (*p* < 0.5)	[47]
Mouse BALB/c eight-weeks-old		28.7	36.2	20 µg	PVP	22.1	Flow cytometry	IV 5 µg/mL/ during 24 h	No effect observed	[48]
Rats (male) Wistar six-weeks-old	ND	ND	ND	ND	ND	ND	MN Bone marrow	Inhalation/4 h per day/200 parts per billion/5 days	Cytotoxicity and genotoxicity	[49]
Rats Sprage-Dawley	Spheroidal	6.3–629	1000	60ng/mL	ND	−22, 26.4, and 29.8	MN Bone marrow Chromosome aberration test	IV 0.5 mg/kg 0.003 mg/kg	No effect observed	[50]
Mouse (male and female) CD1 Five–six-weeks-old	Spheroidal	20.7 ± 3.0	23.5 ±0.7	ND	ND	−16	Comet assay MN lymphocytes from murine spleen	OA 50, 150, and 300 mg/kg/per day/3 days	Neither oxidative damage nor MN frequency increase	[51]

ND: no data; PVP: polyvinylpyrrolidone; IV: intravenous injection; OA: oral administration.

## Data Availability

Data presented in this article is available on request from the corresponding author.

## Supplementary Materials

The following are available online at https://www.mdpi.com/article/10.3390/nano11082096/s1, Table S1: Micronucleated erythrocytes (MN/10,000 TE) in different studied groups, Table S2: Polychromatic erythrocytes in 1000 total erythrocytes (PCE/1000 TE), Table S3: Micronucleated polychromatic erythrocytes in 1000 polychromatic erythrocytes (MNPCE/1000 PCE).
