# Supplementary material for: AgNPs Argovit™ Modulates Cyclophosphamide-Induced Genotoxicity on Peripheral Blood Erythrocytes In Vivo
Source: Nanomaterials (Basel). 2021 Aug 18;11(8):2096. doi: 10.3390/nano11082096 (PMC8399516; doi:10.3390/nano11082096)
Supplement: Supplementary file 1 [file nanomaterials-11-02096-s001.zip › nanomaterials-1314245-supplementary.pdf]

nanomaterials

Supplementary Material

AgNPs Argovit™ Modulates Cyclophosphamide-Induced Genotoxicity on Peripheral Blood Erythrocytes In Vivo

Idalia Yazmin Castañeda-Yslas [1], Olivia Torres-Bugarín [2], Juan Carlos García-Ramos [3,*], Yanis Toledano-Magaña [3], Patricia Radilla-Chávez [3], Nina Bogdanchikova [4], Alexey Pestryakov [5], Balam Ruiz-Ruiz [6] and María Evarista Arellano-García [7,*]

1 Programa de Maestría y Doctorado en Ciencias e Ingeniería (MyDCI). Facultad de Ciencias, Universidad Autónoma de Baja California, Ensenada 22860, Baja California, Mexico; idalia.castaneda@uabc.edu.mx
2 Departamento de Ciclo de vida y Medicina Interna II. Decanato Ciencias de la Salud, Universidad Autónoma de Guadalajara, Zapopan 45129, Jalisco, Mexico; oliviatorres@hotmail.com
3 Escuela de Ciencias de la Salud Unidad Valle Dorado, Universidad Autónoma de Baja California, Ensenada 22890, Baja California, Mexico; yanis.toledano@uabc.edu.mx (Y.T.-M.); patyradilla@uabc.edu.mx (P.R.-C.)
4 Centro de Nanociencias y Nanotecnología, Universidad Nacional Autónoma de México, Ensenada 22860, Baja California, Mexico; nina@cnyn.unam.mx
5 Research School of Chemistry and Applied Biomedical Sciences, Tomsk Polytechnic University, 634050 Tomsk, Russia; pestryakov2005@yandex.ru
6 Departamento de Ciencias de la Salud, Unidad Regional Los Mochis, Universidad Autónoma de Occidente, Los Mochis 81223, Sinaloa, Mexico; balam.ruiz@uadeo.mx
7 Facultad de Ciencias, Universidad Autónoma de Baja California, Ensenada 22860, Baja California, Mexico;
* Correspondence: juan.carlos.garcia.ramos@uabc.edu.mx (J.C.G.-R.); evarista.arellano@uabc.edu.mx (M.E.A.-G.)

Citation: Castañeda-Yslas, I.Y.; Torres-Bugarín, O.; García-Ramos, J.C.; Toledano-Magaña, Y.; Radilla-Chávez, P.; Bogdanchikova, N.; Pestryakov, A.; Ruiz-Ruiz, B.; Arellano-Garcia, M.E. AgNPs Argovit™ Modulates Cyclophosphamide-Induced Genotoxicity on Peripheral Blood Erythrocytes In Vivo. *Nanomaterials* **2021**, *11*, x. https://doi.org/10.3390/nano11082096

Academic Editor(s): Alexandru Mihai Grumezescu; Oana Gherasim

Received: 9 July 2021
Accepted: 13 August 2021
Published: 18 August 2021

Publisher's Note: MDPI stays neutral with regard to jurisdictional claims in published maps and institutional affiliations.

Copyright: © 2021 by the authors. Submitted for possible open access publication under the terms and conditions of the Creative Commons Attribution (CC BY) license (http://creativecommons.org/licenses/by/4.0/).

Average, SD, and statistical significance of PCE/1,000 TE, MNE/10,000 TE, and MNPCE/1,000 PCE values for the different groups are shown in Tables S1-S3.

Table S1. Micronucleated erythrocytes (MN/10,000 TE) in different studied groups.

		Group 1		Group 2		Group 3		Group 4		Group 5	
Hours	*n*	Mean	(±)	Mean	(±)	Mean	(±)	Mean	(±)	Mean	(±)
0	30	10.3	8.0	10.3	8.0	10.3	8.0	10.3	8.0	10.3	8.0
24	6	19.8	6.4	9.8	2.5	39.9	5.0	3.3	1.1	21.3	4.6
48	6	12.9	8.4	22.9	6.3	42.1	6.6	12.7	3.4	13.7	2.3
72	6	18.2	5.8	3.4	0.7	22.7	4.4	6.3	1.7	20.9	5.3
96	6	16.3	6.9	14.4	7.9	21.3	2.9	2.5	1.2	23.4	2.9
120	6	9.6	6.3	14.3	3.1	45.5	7.7	10.3	4.4	67.7	1.4
144	6	7.3	4.2	22.6	3.0	38.2	4.5	20.0	2.2	21.1	4.2
168	6	15.3	4.6	14.1	2.2	27.5	3.7	1.3	1.6	21.3	2.1

n = sample size, mean and standard deviation. Polychromatic erythrocytes in 1000 erythrocytes. Group 1: Water (0, 24, and 48 h); Group 2: CP (0 h); Group 3: AgNPs (0, 24, and 48h); Group 4: AgNPs + CP; CP (0 h) + AgNPs (24, 48, and 72 h); Group 5: AgNPs + CP; CP (0, 48, and 96 h) + AgNPs (24, 72, and 120 h).

Table S2. Micronucleated Polychromatic Erythrocytes in 1000 Polychromatic Erythrocytes (MNPCE/1000 PCE).

		Group 1		Group 2		Group 3		Group 4		Group 5	
Hours	*n*	Mean	(±)	Mean	(±)	Mean	(±)	Mean	(±)	Mean	(±)
0	30	1.6	1.8	1.6	1.8	1.6	1.8	1.6	1.8	1.6	1.8
24	6	2.9	2.6	1.1	1.0	3.7	1.6	2.5	0.6	3.5	0.5
48	6	4.3	3.1	19.7	6.4	3.1	0.4	5.7	1.4	10.1	3.9
72	6	2.5	2.1	3.4	1.7	2.2	1.0	1.2	1.7	4.2	0.9
96	6	0.6	0.7	0.0	0.0	1.6	1.2	1.2	1.3	0.0	0.0
120	6	0.8	0.8	0.6	0.3	4.6	2.8	1.0	1.6	41.5	6.5
144	6	6.0	6.0	2.9	0.8	1.9	0.6	2.9	0.4	21.7	3.1
168	6	0.7	0.8	0.0	0.0	1.2	1.0	0.0	0.0	5.4	1.1

n = sample size, mean and standard deviation. Polychromatic erythrocytes in 1000 erythrocytes. Group 1: Water (0, 24, and 48 h); Group 2: CP (0 h); Group 3: AgNPs (0, 24, and 48h); Group 4: AgNPs + CP; CP (0 h) + AgNPs (24, 48, and 72 h); Group 5: AgNPs + CP; CP (0, 48, and 96 h) + AgNPs (24, 72, and 120 h).

Table S3. Polychromatic Erythrocytes in 1000 Total Erythrocytes (PCE/1000 TE).

		Group 1		Group 2		Group 3		Group 4		Group 5	
Hours	*n*	Mean	(±)	Mean	(±)	Mean	(±)	Mean	(±)	Mean	(±)
0	30	211.0	79.0	211.0	79.0	211.0	79.0	211.0	79.0	211.0	79.0
24	6	247.3	16.3	213.1	27.8	243.2	47.4	285.0	17.7	192.0	4.9
48	6	248.9	60.9	174.1	21.4	225.8	12.6	254.5	67.8	138.2	12.6
72	6	267.2	74.2	84.2	6.1	279.8	8.2	110.4	19.4	69.4	3.7
96	6	217.5	62.0	78.7	20.6	265.8	37.5	136.6	43.5	34.0	2.4
120	6	270.0	71.2	194.4	5.5	166.8	21.0	213.4	21.4	48.5	7.0
144	6	255.2	45.8	188.5	30.8	154.3	18.3	159.5	10.4	66.3	18.2
168	6	272.8	43.4	169.4	25.9	197.7	56.7	247.7	48.4	41.3	3.6

n = sample size, mean and standard deviation. Polychromatic erythrocytes in 1000 erythrocytes. Group 1: Water (0, 24, and 48 h); Group 2: CP (0 h); Group 3: AgNPs (0, 24, and 48h); Group 4: AgNPs + CP; CP (0 h) + AgNPs (24, 48, and 72 h); Group 5: AgNPs + CP; CP (0, 48, and 96 h) + AgNPs (24, 72, and 120 h).
